# Discriminant Analysis of the Geographical Origin of Asian Red Pepper Powders Using Second-Derivative FT-IR Spectroscopy

**DOI:** 10.3390/foods10051034

**Published:** 2021-05-10

**Authors:** Miso Kim, Junyoung Hong, Dongwon Lee, Sohyun Kim, Hyang Sook Chun, Yoon-Ho Cho, Byung Hee Kim, Sangdoo Ahn

**Affiliations:** 1 Department of Chemistry, Chung-Ang University, Seoul 06974, Korea; rlaalth1328@naver.com (M.K.); hjuny94@hanmail.net (J.H.); idleplanet@naver.com (D.L.); dragon725@naver.com (S.K.); 2 Department of Food Science & Technology, Chung-Ang University, Ansung 17546, Korea; hschun@cau.ac.kr; 3 Department of Civil and Environmental Engineering, Chung-Ang University, Seoul 06974, Korea; yhcho@cau.ac.kr; 4 Department of Food and Nutrition, Sookmyung Women’s University, Seoul 04310, Korea; bhkim@sookmyung.ac.kr

**Keywords:** fourier-transform infrared (FT-IR) spectroscopy, second-derivative spectrum, red pepper powder, geographical origin, discriminant analysis

## Abstract

This study aimed to discriminate between the geographical origins of Asian red pepper powders distributed in Korea using Fourier-transform infrared (FT-IR) spectroscopy coupled with multivariate statistical analyses. Second-derivative spectral data were obtained from a total of 105 red pepper powder samples, 86 of which were used for statistical analysis, and the remaining 19 were used for blind testing. A one-way analysis of variance (ANOVA) test confirmed that eight peak variables exhibited significant origin-dependent differences, and the canonical discriminant functions derived from these variables were used to correctly classify all the red pepper powder samples based on their origins. The applicability of the canonical discriminant functions was examined by performing a blind test wherein the origins of 19 new red pepper powder samples were correctly classified. For simplicity, the four most significant variables were selected as discriminant indicator variables, and the applicable range for each indicator variable was set for each geographical origin. By applying the indicator variable ranges, the origins of the red pepper powders of all the statistical and blind samples were correctly identified. The study findings indicate the feasibility of using FT-IR spectroscopy in combination with multivariate analysis for identifying the geographical origins of red pepper powders.

## 1. Introduction

Red peppers (*Capsicum annuum L*.) are perennial plants of the family Solanaceae and are widely grown worldwide. The capsaicinoids specifically contained in red peppers are pungent alkaloids and are known to promote energy metabolism [1]. In addition, carotenoids and vitamin C, which are abundant in red peppers, have been reported to have anti-cancer effects [2,3]. Red peppers are mainly used for their hot spicy flavor and red color. They are predominantly processed into a dried powder form for easy transport to markets worldwide. The quality and cost of red pepper powders vary considerably depending on their country of origin. For instance, the quality of imported peppers is reduced owing to freezing or other pretreatment processes [4]. Consequently, consumers typically prefer domestic products [5]. In some cases, retailers deceive consumers by omitting the country of origin of the red pepper powders to inflate their margins [6]. Therefore, it is necessary to develop an accurate and rapid method for identifying the origin of red pepper powders. 

Several factors contribute to the differences between plants of different geographical origins [7,8]. Each country has a different crop cultivation environment, such as soil and climate, which lead to differences in the metabolite compositions of plants. Therefore, many studies have been conducted to discriminate foods and agricultural products based on their geographical origins by analyzing their metabolite profiles using mass spectrometry (MS) [9], nuclear magnetic resonance (NMR) spectroscopy [10,11], and chromatography [12]. Recently, simple non-destructive Fourier-transform infrared (FT-IR) spectroscopic techniques have been used for metabolite research [13,14]. The FT-IR method has been applied in combination with multivariate statistical analysis for the geographical discrimination of Korean and Chinese soybeans [15], European saffron [16], European olive oils [17], and European and South American honey [18]. FT-IR, which provides a variety of information on chemical bonds and functional groups, involves the use of inexpensive equipment and does not require the special pretreatment of samples regardless of their state. Diverse FT-IR information has also been used to study the adulteration and authenticity of foods [19]. Moreover, recent studies have combined FT-IR with other instrumental analysis methods to improve the efficiency of food analysis [20,21]; in particular, multivariate statistical analyses have been applied to effectively process instrumental analytical data, requiring the observation of minute differences in the compositions or spectral profiles of various metabolites [22].

The geographical classification of Asian red peppers has also been studied [23,24,25,26]. Yin et al. proposed a simple method to determine the geographical origins of red peppers from various regions in China through multivariate analysis of sensory characteristics, such as color, taste, and smell [24]. Zhang et al. conducted a study to effectively distinguish the regions of origin of red peppers in China by analyzing the multi-elements obtained using the inductively coupled plasma (ICP) methods with various chemometric tools [25]. Song et al. demonstrated the possibility of discriminating the geographical origins of Korean red peppers through multivariate statistical analysis of the stable isotope ratio (^87^Sr/^86^Sr) affected by cultivation environments [26]. Recently, it has been demonstrated that ^1^H NMR spectroscopy, combined with multivariate statistical analyses, has a significant predictive potential for identifying the geographical origins of Asian red pepper powders [23]. As is well known, NMR spectroscopy is an extremely beneficial analytical method for metabolite studies because NMR measurements are highly reproducible and can provide both qualitative and quantitative information simultaneously. However, NMR spectroscopy incurs a high instrumental cost and requires pretreatment processes to extract the metabolite components from solid samples, such as red pepper powders.

In this study, FT-IR spectroscopy was used to develop an alternative, simple, and convenient experimental method combined with multivariate statistical analysis for determining the geographical origins of red pepper powders from Korea, China, and Vietnam. The second derivative of the FT-IR spectrum was used to enhance the resolution of broadly overlapping peaks and improve peak quantification by removing baseline errors [27,28,29,30]. A one-way analysis of variance (ANOVA) test was used to identify whether the peak variables were significantly different depending on their origins [31]. Canonical discriminant analysis, as a multivariate statistical analysis, was used to obtain the discriminant functions for classifying the geographical origins of red pepper powder samples [32]. The obtained canonical discriminant functions represent linear combinations of the meaningful variables identified by the ANOVA test, and exhibit the highest possible multiple correlations with the origins. The applicability of the discriminant functions was verified through a blind test. Additionally, to easily discriminate new red pepper samples based on their geographical origins without complicated statistical processes, several variables having significant influences on the discrimination were selected as the discriminant indicator variables, and their applicable ranges were set for each geographical origin. The feasibility of this method was also verified by using it to correctly identify the origins of the blind samples. When appropriate discriminant indicator variables and ranges are set, the application becomes very simple. Thus, the proposed method may be considered as a more effective and convenient method for discriminating new samples in comparison with other discrimination methods that require statistical processing.

## 2. Materials and Methods

### 2.1. Red Pepper Powder Samples

A total of 105 Asian (Korean, Chinese, and Vietnamese) red pepper powders (or dried red peppers) distributed in Korea were collected as samples. Korean red pepper powders were obtained from local producers or reliable suppliers, such as agricultural cooperatives. Chinese and Vietnamese red pepper powders imported to Korea, through the Korea Agro-Fisheries Trade Corporation, were purchased from local markets. Among the total samples, 86 red pepper powders (Korean = 50, Chinese = 23, Vietnamese = 13) were used for statistical analyses to establish the discriminant functions and indicator variables, which could be used to distinguish their geographical origins. The remaining 19 red pepper powders (Korean = 9, Chinese = 5, Vietnamese = 5) were used as blind test samples to verify the applicability of the established discriminant functions and indicator variables. All the dried red pepper powder samples were stored in a refrigerator at 4 °C. However, commonly sold red pepper powders are mixtures of peel and seed fragments with a length of 1–3 mm, making it difficult to reflect all the component information in the FT-IR spectra and ensure reproducibility of measurements. Therefore, the powder was further ground into a fine powder (with particle diameters of ≤ 200 μm) in a food grinder before measurement. The prepared fine powder samples exhibited good reproducibility in repeated measurements. Three Korean samples purchased in the form of dried red pepper were first ground into powders using a crusher, and then further ground into finer powders as in the other samples.

### 2.2. FT-IR Measurement

Finely ground red pepper powder samples were loaded onto an FT-IR spectrometer (TENSOR-27; Bruker Optics GmbH, Karlsruhe, Germany) equipped with a diamond attenuated total reflectance (ATR) accessory (A225/Q Platinum ATR; Bruker Optics GmbH, Karlsruhe, Germany). All the spectra were acquired in absorbance mode, in the wavenumber range of 4000–400 cm^−1^, with 32 repeated scans and a resolution of 4 cm^−1^. The acquisition time was less than 1 min for each measurement. To ensure the representativeness and reproducibility of the obtained FT-IR spectra, measurements were repeated five times for each sample and statistically averaged. The ATR crystal was cleaned with ethyl alcohol before every measurement. Atmospheric correction was also performed for each measurement to eliminate the effects of CO_2_ and H_2_O in the atmosphere. OMINC software (version 8.2, ThermoFisher Scientific Inc. Waltham, MA, USA) was used to process the obtained spectra of the red pepper powder samples. Second derivatives of the processed FT-IR absorbance spectra were derived using the Savitzky–Golay (SG) numerical algorithm with third-order polynomials at seven smoothing points [33,34,35]. The SG method is a commonly used filtering and smoothing technique to remove background effects and any possible noise in the spectrum during second-order differentiation. Through the differentiation process, the sensitivity and resolution of the spectrum were improved by correcting the baseline drift and separating the overlapped peaks [33,34,35]. The normalized value of each peak in the second-derivative spectra was used for statistical analyses to establish the origin discriminant functions and indicator variables.

### 2.3. Multivariate Statistical Analysis

Statistical analyses for the second-derivative FT-IR spectral data were performed using IBM SPSS Statistics software (version 26, SPSS Inc., Chicago, IL, USA). Tests of homogeneity of variance were conducted to determine if each peak variable was equally distributed according to the origin group. For the variables with equal variance, a one-way ANOVA test was used to determine significant differences in the peak variables depending on their origin group (significance level, *p* < 0.05). Canonical discriminant analyses were performed with the selected variables to determine the discriminant functions capable of effectively classifying the geographical origins of the red pepper powder samples. Additionally, by selecting several indicator variables that contribute significantly to the discriminant functions and setting the ranges of their values, we determined whether the geographical origin could be easily identified without the statistical dataset. The applicability of both the discriminant functions and the indicator variables obtained were tested through a blind test [28,36,37,38,39].

## 3. Results and Discussion

### 3.1. FT-IR Spectrum of Red Pepper Powder

**Figure 1:**
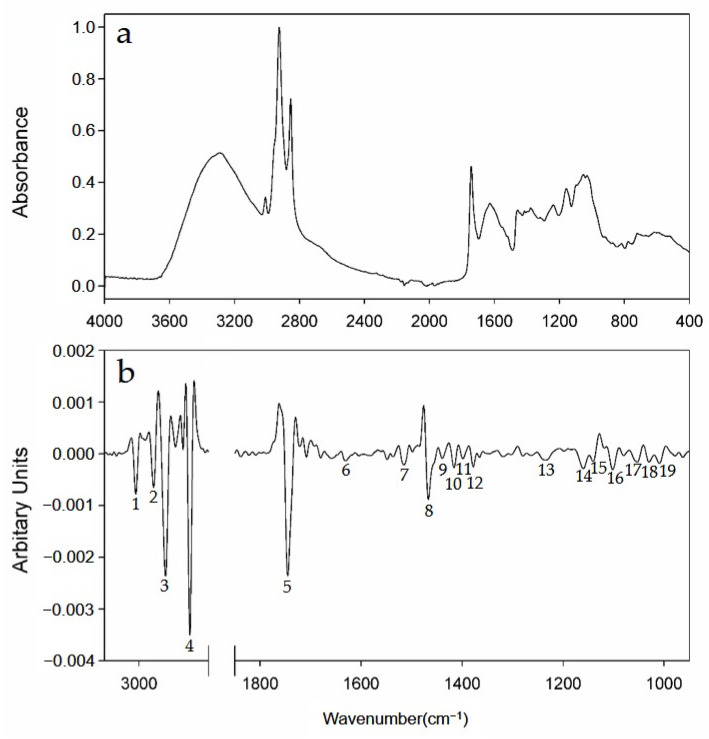
Representative (**a**) FT-IR absorption spectrum of a Korean red pepper powder sample, and (**b**) its second-derivative spectrum (partially expanded at the variables) with numbered peaks (corresponding to the variables in Table 1).

Figure 1 illustrates a representative FT-IR spectrum of a red pepper powder sample and its second-derivative spectrum. Using the second derivative of the FT-IR spectrum, more sophisticated spectral data were obtained, while broadly overlapping peaks in the original absorption spectrum could be isolated. The second-derivative process also improved the peak quantification by removing the baseline errors. As summarized in Table 1, 19 distinguishable peaks were selected and labeled in the second-derivative FT-IR spectrum. The peaks were assigned by referring to previous studies [15,16,28,40,41,42,43,44,45].

The broad band at approximately 3400 cm^−1^ is mainly due to the stretching of the O–H bonds, because red pepper powder has a low protein content [7] and easily absorbs moisture [46]. The peak at 3010 cm^−1^ (P1) is attributed to sp^2^ C–H stretching, while the peaks at 2958 cm^−1^, 2924 cm^−1^, and 2852 cm^−1^ (P2, P3, P4) are attributed to the sp^3^ C–H stretching of metabolites in the red pepper powders. The strong peak at 1745 cm^−1^ (P5) is due to the C=O stretching, and the weak peak at 1653 cm^−1^ (P6) is due to the C=C stretching. The aromatic C-C stretching band appears at 1516 cm^−1^ (P7), and several C-H bending bands appear at 1516–1238 cm^−1^ (P8–P13). The various C–O stretching bands of the ester and ether groups appear at 1238–1008 cm^−1^ (P3–P19) which are mainly attributed to the lipids and carbohydrates in the red pepper powders [43,45]. The intensities of the peaks differ slightly depending on the distribution of various metabolites in the red pepper powders. Hence, the statistical analysis of this information could be used to discriminate between the geographical origins. For further statistical analysis, the absolute peak values normalized by the intensity of the C–O stretching peak at 1008 cm^−1^ were used.

### 3.2. Statistical Analysis

#### 3.2.1. Canonical Discriminant Analysis

Canonical discriminant analysis was performed as a multivariate statistical analysis to achieve the most discriminative peak variables for the arrangement of red pepper powder samples in a lower dimensional space by maximizing the distances between the origin groups. To ensure the robustness of these statistical processes, the homogeneity of the variance of each variable must be considered [31]. Therefore, to select suitable variables for the statistical analysis, a variance homogeneity test was conducted first. As a result of testing 18 peaks, it was confirmed that eight peak variables, namely P5, P7, P8, P10, P12, P14, P16, and P17, had equal variance (*p* > 0.05), while the 10 remaining peaks did not exhibit equal variance (*p* < 0.05) (Table S1).

In this study, an ANOVA test was performed to determine the second-derivative FT-IR peak variables with meaningful differences among the Korean, Chinese, and Vietnamese red pepper powder groups. The ANOVA test verified the equality of the group means of variables using the *F* test, and determined whether the means of three or more groups were different [31]. Since the ANOVA test is a parametric test, only the eight peaks with equal variance identified in the previous test of homogeneity of variance were considered [31]. All the eight peak variables exhibited significant differences in the origins (*p* < 0.001) with large *F* values (Table 2). As can be seen in Table 2, a smaller Wilks’ lambda value (i.e., a larger *F*-value) implies a higher significance in the discrimination analysis.

**Figure 2:**
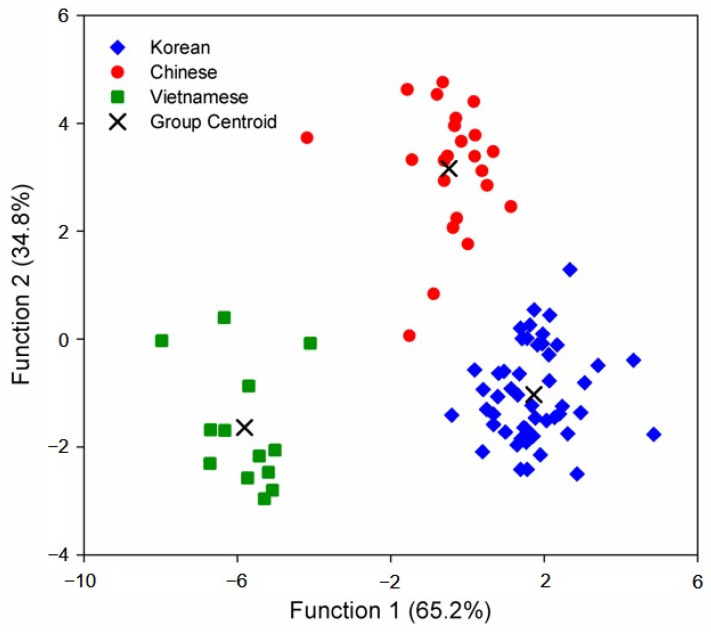
Scatter plot of two discriminant scores for the geographic origins of Korean, Chinese, and Vietnamese red pepper powders.

These eight significant variables were used for the canonical discriminant analysis to establish the discriminant functions. Two canonical discriminant functions were derived for identifying the red pepper powder samples from different origins, and accounted for 100% of the variance. Functions 1 and 2 accounted for 65.2% and 34.8% of the total variance, respectively. The separation between the red pepper powder samples of different geographical origins in the discriminant space was investigated by scatter plotting the discriminant function scores. The score plot showed good separation among the samples from three different origins (Figure 2), suggesting that the variables used to derive the discriminant functions provided sufficient information to identify the geographical origins of red pepper powders. The Korean and Vietnamese samples were found to be completely distinguishable from each other, while the Chinese samples appeared relatively widely scattered between the Korean and Vietnamese samples. This may be attributed to the diversity of the Chinese samples, reflecting the characteristics of China’s large geographical area. 

To verify and examine the predictive discrimination capability of the established canonical discriminant functions, we reclassified the red pepper powder samples used in the multivariate statistical analysis, according to their geographical origins. Table 3 indicates that the canonical discriminant functions correctly classified all 86 red pepper powder samples (50 Korean, 23 Chinese, and 13 Vietnamese) according to their geographical origins (100% of the original group cases were correctly classified), while only one Chinese sample was incorrectly classified in the cross-validation (98.8% of the original group cases were correctly classified). These results were similar to the discrimination results of the origins of 62 Asian red pepper powder (36 Korean, 17 Chinese, and 9 Vietnamese) samples using ^1^H NMR spectroscopy [23]. In particular, this result was of significance considering that various metabolite components even with minor contents could be used as individual indicators in the ^1^H NMR analysis. By comparing the analysis results of the mineral elements [25] and sensor characteristics [24] of red peppers from other regions in China using various multivariate statistical analysis methods, it can be observed that their regional scopes were different. However, it can be confirmed that the second-derivative FT-IR method can be sufficiently utilized to discriminate the origins of red pepper powders. In addition, similar discrimination abilities can be confirmed by comparing previous results of the origins of other foods, such as olive oil and honey, using the FT-IR technique [17,18]. Overall, these results indicate that second-derivative FT-IR spectroscopy combined with canonical discriminant analysis has the potential to discriminate Asian red pepper powders according to their geographical origins.

#### 3.2.2. Discriminant Indicator Variables

It was confirmed that Asian red pepper powders could be effectively discriminated according to their geographical origins by canonical discriminant analysis of the signals obtained from the second-derivative FT-IR spectra. This protocol can also be applied to the discrimination of new red pepper powder samples through statistical processes. If several indicator variables suitable for discriminating the origin of red pepper samples are selected and appropriate ranges are set for them, rapid and facile discrimination of the geographical origins of new red pepper powder samples is possible without the need for a specific statistical program or process.

The Pearson coefficients are summarized in the structure matrix table (Table 4), which shows the correlation of each variable with each canonical discriminant function [47,48,49]. This table reveals that P12 and P17 are the most significant variables in discriminant Functions 1 and 2 (with correlations of −0.475 and 0.714), respectively. P14, and P8 also show high significance in both functions.

**Figure 3:**
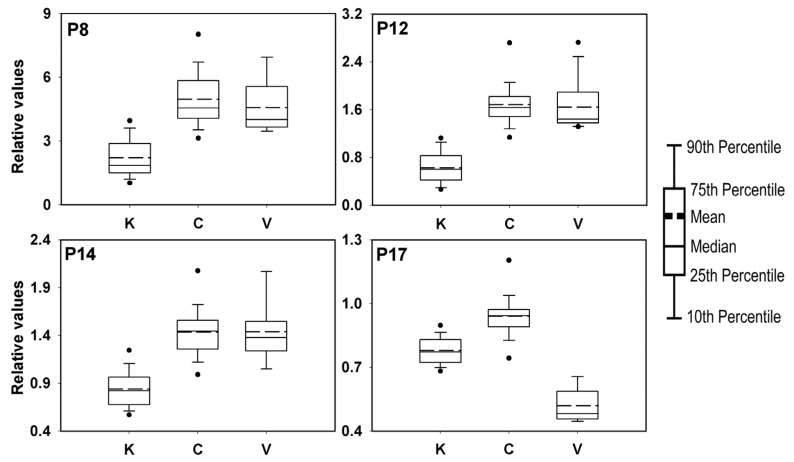
Box plots for variables that are highly correlated with the canonical discriminant functions (K = Korean, C = Chinese, and V = Vietnamese). The dots indicate the 5th and 95th percentiles.

These four peak variables (P8, P12, P14, and P17) were also found to have high significance in the mean difference, with an *F*-value of 60 or more in the one-way ANOVA test (Table 2). The distribution of data between the geographical origin groups of these four variables were compared as box plots (Figure 3), confirming that P12 and P17 were the most effective variables for discriminating the Korean and Vietnamese samples, respectively, from those of other geographical origins. Additionally, the distribution characteristics of P8, P12, and P14 were similar, whereas those for P17 were different. This was also confirmed in the Pearson correlation matrix, which shows the correlations among variables (Table S2).

Considering their correlation with the discriminant functions, mean difference, and difference in distribution values, P8 and P14, along with the most significant variables P12 and P17, were selected as indicator variables for discriminating the origins of Asian red pepper powder samples. To discriminate the geographical origins using the specific indicator variables, they must have ranges differentiated according to the origins.

For the Korean red pepper samples, the distribution values of P8 and P12 were smaller than those of the others. These signals can be attributed to C–H stretching vibrations, which are derived from various metabolites containing alkyl groups, and are likely largely influenced by the hydrocarbon chains of fatty acids. Because the fatty acid content is relatively higher in seeds than in the peel of red pepper [50], it can be estimated that the Korean red pepper powder samples contain relatively fewer seeds than the Chinese or Vietnamese samples. Moreover, the P17 signal attributed to the C–O stretching vibration arising mainly from the fructosyl unit [45] was observed to be small in the Vietnamese samples. This implies that the Vietnamese red pepper powders had relatively lower fructose content than those of the Korean and Chinese peppers, which was also confirmed in previous NMR experiments (Figure S1) [23]. For the Chinese red pepper powder samples, all four variables exhibited relatively higher means than the others. However, owing to the diversity of the Chinese samples, the ranges of all the indicator variables significantly overlapped with the ranges of those for other origins; hence, establishing independent variable ranges for Chinese samples was not possible.

Based on these observations, the ranges of the discriminant indicator variables that could discriminate between Korean and Vietnamese red pepper powder samples were set as presented in Table 5. 

The range of each discriminant variable was set based on their maximum or minimum values, or by considering values between the minimum and maximum based on the relative distribution characteristics of each variable value [27,37,38]. For example, in the case of the P8 variable, because Korean red pepper powders had the lowest distribution, its range was set below the maximum value for Korean samples. On the contrary, the Vietnamese samples had a relatively high distribution and, thus, were set above the minimum value for Vietnamese samples. It is worth noting that if each variable value obtained the analysis of more samples satisfied the normal distribution sufficiently, the ranges could be established using a statistical technique as well.

To confirm the suitability of the selected indicator variables and their range settings, we reclassified the red pepper powder samples used in the multivariate statistical analysis, based on their geographical origins. A sample was attributed to a specific origin only if the values of all the indicator variables for the sample were within the discriminant ranges for that origin; the results are summarized in Table 6. When the ranges of the indicator variables for the Korean red pepper powder samples were applied, all 50 Korean samples were identified as “Korean,” and the remaining 36 samples (23 Chinese and 13 Vietnamese) were all classified as “not Korean.” When applying the ranges of the indicator variables for the Vietnamese red pepper powder samples to the 36 “not Korean” samples, all 13 Vietnamese samples were identified as “Vietnamese” and the remaining 23 Chinese samples were identified as “not Vietnamese.” Changing the order of applying the indicator variable ranges for the Korean and Vietnamese samples produced the same results, indicating that the two sets of ranges were well separated.

Setting the range of discriminant indicator variables aids in determining the authenticity of food, based on the content of intrinsic ingredients (such as metabolites and minerals) [28,37,38,39]. However, it is not easy to apply this method to discriminate between the origins of the same food. Therefore, it is meaningful that the geographical origin was correctly classified by setting several discriminant indicators and their ranges. Recently, FT-IR spectroscopy combined with statistical analysis has been actively applied to determine the authenticity, adulteration, and geographical origins of various foods. If the discriminant indicator variables and their ranges are set suitably, more effective and practical use of such results can be realized.

### 3.3. Blind Tests

To evaluate the applicability of the developed statistical discrimination method and the discriminant indicator variables to new samples, a blind test was performed on 19 new red pepper powder samples (9 Korean, 5 Chinese, and 5 Vietnamese), which were not used in the previous statistical analyses. The geographical origins were correctly classified for all the 19 blind red pepper powder samples using the established canonical discriminant functions (Table 7).

Table 8 presents the classification results of comparing the values of the indicator peak variables obtained from the second-derivative FT-IR spectra of the blind samples with the discriminant ranges for the Korean and Vietnamese red peppers. When the ranges of the indicator variables for the Korean red pepper powder were applied, nine blind samples were correctly identified as “Korean”, and the remaining 10 blind samples were classified as “not Korean”. When applying the ranges of indicator variables for Vietnamese pepper to 10 blind “not Korean” samples, five samples were correctly identified as “Vietnamese”. As in the canonical discriminant analysis, the other five samples that were classified as neither Korean nor Vietnamese can be assumed to be Chinese red pepper powder samples. These results indicate that the indicator ranges can be conveniently used to classify the geographical origins of new red pepper powder samples, even if they are established using a limited number of samples.

## 4. Conclusions

In this study, we investigated the feasibility of second-derivative FT-IR spectroscopy, combined with multivariate statistical analysis, to discriminate red pepper samples from Korea, China, and Vietnam, based on their geographical origins. Canonical discriminant functions for classifying Asian red pepper powders based on geographical origins were derived from the discriminant analysis, and the discriminating capability of the functions was verified by 100% correct reclassification of the origins of the powder samples used in the analysis. The results of the blind test to classify new red pepper powder samples according to geographical origins confirmed that the derived discriminant functions could correctly classify all new test samples. Although the classification method using the canonical discrimination functions is highly accurate, it requires the statistical data and program used to create the functions to discriminate the origins of new samples. To compensate for these limitations and simply determine the geographical origin without a special statistical program, four indicator variables with large differences in values according to their origins were selected from the variables used in the statistical analysis, and their origin-specific ranges were set. These indicator ranges were successfully used to correctly classify the geographical origins of all statistical samples and blind samples. Although applied to a limited number of samples, the use of the ranges of discriminant indicator variables provides a simple classification method for new samples. Further analyses of more red pepper powder samples, including samples from other countries, may enhance the capability and accuracy of the method of using both the canonical discriminant functions and the discriminant indicator variable ranges. In addition, the discriminant method that uses set ranges of the discriminant indicator variables may be useful in terms of experimental methodology; however, it can be expected to have more applications useful in fields that manage the traceability of foods.

In conclusion, the findings of this study indicate that the second-derivative FT-IR spectroscopy is a reliable, low-cost, and convenient analytical method for discriminating Asian red pepper powders according to their geographical origins.

## Figures and Tables

**Table 1:** Assignment of peaks in the second-derivative FT-IR spectrum of red pepper powders.

Variable	Wavenumber (cm^−1^)	Functional Group	Mode of Vibration
P1	3010	=C–H (*cis*-)	Stretching [40,41]
P2	2958	–C–H (CH_3_)	Stretching [40,41]
P3	2924	–C–H (*asym* CH_2_)	Stretching [40,41,44]
P4	2852	–C–H (*sym* CH_2_)	Stretching [40,41,44]
P5	1745	–C=O (ester)	Stretching [40,41,44]
P6	1653	–C=C– (*cis*-)	Stretching [40,41]
P7	1516	–C–C– (aromatic)	Stretching [42]
P8	1468	–C–H (CH_2_, CH_3_)	Bending [40,41,44]
P9	1439	–C–H (CH_2_)	Bending [45]
P10	1415	=C–H (cis-)	Bending [28,41,42]
P11	1398	–C–H (CH_2_, CH_3_)	Bending [15,16,41]
P12	1377	–C–H (CH_3_)	Bending [16,40,41,44]
P13	1238	–C–O (ester), –C–H (CH_2_)	Stretching, Bending [41,44]
P14	1159	–C–O (ester)	Stretching [40,41,44,45]
P15	1142	–C–O	Stretching [43,45]
P16	1101	–C–O	Stretching [43,45]
P17	1053	–C–O	Stretching [43,45]
P18	1028	–C–O	Stretching [43,45]
P19	1008	–C–O	Stretching [43,45]

**Table 2:** Tests of equality of group means.

Variable	Wilks’ Lambda	*F*	df_1_	df_2_	Significance Level, *p*
P05	0.480	44.881	2	83	<0.001
P07	0.776	11.946	2	83	<0.001
P08	0.401	61.900	2	83	<0.001
P10	0.550	34.005	2	83	<0.001
P12	0.264	115.938	2	83	<0.001
P14	0.383	66.762	2	83	<0.001
P16	0.623	25.106	2	83	<0.001
P17	0.246	127.075	2	83	<0.001

**Table 3:** Reclassification results for the origins of red pepper powder samples using the canonical discriminant functions.

Origin		Predicted Group			Total	Accuracy (%)
		Korean	Chinese	Vietnamese		
**Original**	Korean	50	0	0	50	100
	Chinese	0	23	0	23	100
	Vietnamese	0	0	13	13	100
**Cross-validated ^a^**	Korean	50	0	0	50	100
	Chinese	1	22	0	23	95.6
	Vietnamese	0	0	13	13	100

^a^ Cross-validation was performed only for those cases in the analysis. In cross-validation, each case is classified using the functions derived from all other cases except that case.

**Table 4:** Structure matrix table with coefficients for the peak variables used in discrimination analysis.

Variable	Structure Matrix	
	Function 1	Function 2
P5	−0.303	0.333
P7	−0.184	0.11
P8	−0.326	0.437
P10	−0.202	0.371
P12	−0.475	0.555
P14	−0.368	0.409
P16	−0.185	0.306
P17	0.394	0.714

**Table 5:** Ranges of the indicator variables for Korean and Vietnamese samples.

Peak No.	Wavelength (cm^−1^)	Vibration	Range	
			Korean	Vietnamese
P8	1468	C–H (CH_2_, CH_3_) bending	<4.945	>3.445
P12	1377	–C–H (CH_3_) stretching	<1.155	>1.305
P14	1159	–C–O (ester) stretching	<1.555	0.985–2.085
P17	1053	–C–O stretching	0.620–0.945	<0.699

**Table 6:** Reclassification results for red pepper powder samples using the ranges of the indicator variables.

Applied Ranges	Origin	Predicted Results			
		Predicted	Not Predicted	Total	Accuracy (%)
Korean	Korean	50	0	50	100
	Not Korean	0	36	36	100
Vietnamese	Vietnamese	13	0	13	100
	Not Vietnamese	13	0	13	100

**Table 7:** Classification of the geographical origins of the blind samples using the established canonical discriminant functions.

Sample No.	Function 1	Function 2	Origin	Predicted	Probability
1	−1.075	−2.451	Korean	Korean	0.998
2	0.995	−0.257	Korean	Korean	0.991
3	2.643	−1.205	Korean	Korean	1
4	2.118	−0.816	Korean	Korean	1
5	1.979	−1.449	Korean	Korean	1
6	1.018	−0.926	Korean	Korean	0.999
7	1.49	−0.881	Korean	Korean	1
8	1.66	0.506	Korean	Korean	0.963
9	0.757	−0.917	Korean	Korean	0.998
10	−0.434	2.797	Chinese	Chinese	1
11	−1.023	2.405	Chinese	Chinese	1
12	−1.402	0.23	Chinese	Chinese	0.946
13	−1.825	7.135	Chinese	Chinese	1
14	−0.862	4.166	Chinese	Chinese	1
15	−5.508	1.616	Vietnamese	Vietnamese	1
16	−4.216	−0.415	Vietnamese	Vietnamese	1
17	−4.529	1.186	Vietnamese	Vietnamese	0.977
18	−5.688	0.018	Vietnamese	Vietnamese	1
19	−7.532	−0.289	Vietnamese	Vietnamese	1

**Table 8:** Classification of the blind samples based on their geographical origins using the ranges of the indicator variables for Korean and Vietnamese samples.

Sample No.	P8	P12	P14	P17	Origin	Predicted
1	2.703	0.804	1.097	0.624	Korean	Korean
2	2.637	0.749	1.081	0.794	Korean	Korean
3	2.379	0.549	0.96	0.858	Korean	Korean
4	1.328	0.414	0.712	0.808	Korean	Korean
5	0.955	0.268	0.577	0.753	Korean	Korean
6	1.562	0.465	0.709	0.729	Korean	Korean
7	1.623	0.522	0.772	0.782	Korean	Korean
8	2.393	0.781	0.912	0.873	Korean	Korean
9	1.903	0.637	0.753	0.752	Korean	Korean
10	4.197	1.525	1.437	0.935	Chinese	(Chinese) *
11	4.544	1.515	1.284	0.841	Chinese	(Chinese) *
12	3.684	1.287	1.4	0.759	Chinese	(Chinese) *
13	7.074	2.778	2.22	1.117	Chinese	(Chinese) *
14	5.164	1.858	1.534	0.921	Chinese	(Chinese) *
15	4.362	1.777	1.648	0.69	Vietnamese	Vietnamese
16	5.107	1.818	1.564	0.604	Vietnamese	Vietnamese
17	5.32	1.919	1.608	0.694	Vietnamese	Vietnamese
18	5.67	2.1	1.824	0.617	Vietnamese	Vietnamese
19	6.971	2.468	1.955	0.559	Vietnamese	Vietnamese

* Samples were classified as neither Korean nor Vietnamese.

## Supplementary Materials

The following are available online at https://www.mdpi.com/article/10.3390/foods10051034/s1, Figure S1: Expanded (fructose hydrogen regions, normalized to the integral sum of all signals) ^1^H NMR spectra of Asian red pepper powders at 600 MHz NMR. Table S1: Test of homogeneity of variance between the groups of Korean, Chinese, and Vietnamese red pepper powders using second derivative values of FT-IR spectra. Table S2: Pearson’s Correlation Matrix.
