# Supplementary material for: Discriminant Analysis of the Geographical Origin of Asian Red Pepper Powders Using Second-Derivative FT-IR Spectroscopy
Source: Foods. 2021 May 10;10(5):1034. doi: 10.3390/foods10051034 (PMC8150797; doi:10.3390/foods10051034)
Supplement: Supplementary file 1 [file foods-10-01034-s001.zip › foods-1186027-supplementary.pdf]

foods

Supplementary Materials

Discriminant Analysis of the Geographical Origin of Asian Red Pepper Powders Using Second-Derivative FT-IR Spectroscopy

Miso Kim [1,†], Junyoung Hong [1,†], Dongwon Lee [1], Sohyun Kim [1], Hyang Sook Chun [2], Yoon-Ho Cho [3], Byung Hee Kim [4] and Sangdoo Ahn [1,*]

[1] Department of Chemistry, Chung-Ang University, Seoul 06974, Republic of Korea; rlaalth1328@naver.com (M.K.), hjuny94@hanmail.net (J.H.), idleplanet@naver.com (D.L.); dragon725@naver.com (S.K.), sangdoo@cau.ac.kr (S.A.)
[2] Department of Food Science & Technology, Chung-Ang University, Ansung 17546, Republic of Korea; hschun@cau.ac.kr (H.S.C.)
[3] Department of Civil and Environmental Engineering, Chung-Ang University, Seoul, 06974, Republic of Korea; yhcho@cau.ac.kr (Y.H.C.)
[4] Department of Food and Nutrition, Sookmyung Women's University, Seoul 04310, Republic of Korea; bhkim@sookmyung.ac.kr (B.H.K.)
* Correspondence: sangdoo@cau.ac.kr (S.A.); Tel.: +82-2-820-5230 (S.A.)
† These authors contributed equally to this work.

Keywords: Fourier-transform infrared (FT-IR) spectroscopy; second-derivative spectrum; red pepper powder; geographical origin; discriminant analysis

Supplementary Materials: The following are available online at www.mdpi.com/2304-8158/10/5/1034/s1, Figure S1: Expanded (fructose hydrogen regions, normalized to the integral sum of all signals) ^{1}H NMR spectra of Asian red pepper powders at 600MHz NMR, Table S1: Test of homogeneity of variance between the groups of Korean, Chinese, and Vietnamese red pepper powders using second derivative values of FT-IR spectra, Table S2: Pearson's Correlation Matrix.

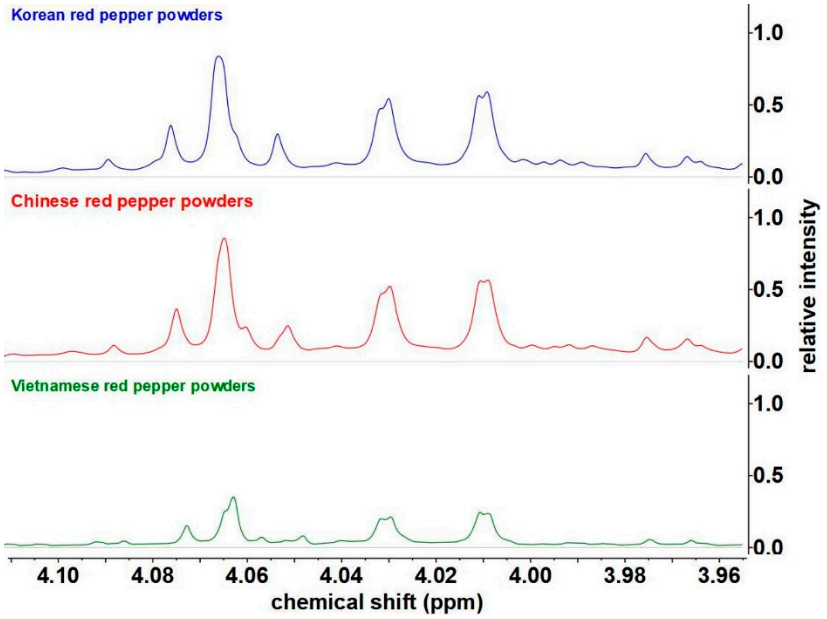

Figure S1: Expanded (fructose hydrogen regions, normalized to the integral sum of all signals) ^{1}H NMR spectra of Asian red pepper powders at 600MHz NMR. [1,2]

Table S1: Test of homogeneity of variance between the groups of Korean, Chinese, and Vietnamese red pepper powders using second derivative values of FT-IR spectra.

Peak No.	Levene statistic	df_1	df_2	Significance level[1]
P1	4.320	2	83	0.016
P2	7.860	2	83	0.001
P3	11.557	2	83	0.000
P4	9.219	2	83	0.000
P5	1.167	2	83	0.316
P6	11.136	2	83	0.000
P7	0.556	2	83	0.576
P8	1.003	2	83	0.371
P9	6.640	2	83	0.002
P10	0.417	2	83	0.660
P11	4.830	2	83	0.010
P12	1.287	2	83	0.281
P13	6.084	2	83	0.003
P14	1.011	2	83	0.368
P15	5.750	2	83	0.005
P16	1.630	2	83	0.202
P17	0.441	2	83	0.645
P18	8.456	2	83	0.000

[1] All results based on mean.

Table S2. Pearson's Correlation Matrix

Pearson Correlation	P17	P16	P14	P12	P10	P8	P7	P5
P17	1	0.182	0.069	0.111	0.242*	0.155	0.051	0.051
P16	0.182	1	0.911**	0.801**	0.764**	0.782**	0.361**	0.794**
P14	0.069	0.911**	1	0.935**	0.841**	0.898**	0.531**	0.886**
P12	0.111	0.801**	0.935**	1	0.888**	0.957**	0.595**	0.916**
P10	0.242*	0.764**	0.841**	0.888**	1	0.954**	0.683**	0.792**
P8	0.155	0.782**	0.898**	0.957**	0.954**	1	0.726**	0.847**
P7	0.051	0.361**	0.531**	0.595**	0.683**	0.726**	1	0.420**
P5	0.051	0.794**	0.886**	0.916**	0.792**	0.847**	0.420**	1

*. Correlation is significant at the 0.05 level (2-tailed).

**. Correlation is significant at the 0.01 level (2-tailed).

References

1. Villa-Ruano, N.; Ramirez-Meraz, M.; Mendez-Aguilar, R.; Zepeda-Vallejo, L.G.; Alvarez-Bravo, A.; Perez-Hernandez, N.; Becerra-Martinez, E. 1H NMR-based metabolomics profiling of ten new races from Capsicum annuum cv. serrano produced in Mexico. *Food Res Int* **2019**, *119*, 785-792, doi:10.1016/j.foodres.2018.10.061.
2. Lee, D.; Kim, M.; Kim, B.H.; Ahn, S. Identification of the Geographical Origin of Asian Red Pepper (Capsicum annuum L.) Powders Using 1H NMR Spectroscopy. *Bulletin of the Korean Chemical Society* **2020**, *41*, 317-322, doi:10.1002/bkcs.11974.
